# Ballistic Behavior of Epoxy Composites Reinforced with Amazon Titica Vine Fibers (*Heteropsis flexuosa*) in Multilayered Armor System and as Stand-Alone Target

**DOI:** 10.3390/polym15173550

**Published:** 2023-08-26

**Authors:** Juliana dos Santos Carneiro da Cunha, Lucio Fabio Cassiano Nascimento, Ulisses Oliveira Costa, Wendell Bruno Almeida Bezerra, Michelle Souza Oliveira, Maria de Fátima Vieira Marques, Ana Paula Senra Soares, Sergio Neves Monteiro

**Affiliations:** 1 Department of Materials Science, Military Institute of Engineering—IME, Praça General Tibúrcio, 80, Urca, Rio de Janeiro 22290-270, RJ, Brazil; lucio@ime.eb.br (L.F.C.N.); ulissesolie@gmail.com (U.O.C.); wendellbez@gmail.com (W.B.A.B.); oliveirasmichelle@ime.eb.br (M.S.O.); snevesmonteiro@gmail.com (S.N.M.); 2 Institute of Macromolecules Professor Eloisa Mano, Federal University of Rio de Janeiro, Horácio Macedo Av., 2.030, Bloco J, University City, Rio de Janeiro 21941-598, RJ, Brazil; fmarques@ima.ufrj.br; 3 Department of Organic Processes, School of Chemistry, Federal University of Rio de Janeiro, Rio de Janeiro 21941-598, RJ, Brazil; soaressana@hotmail.com.br

**Keywords:** natural fiber composite, titica vine fibers, multilayered armor, ballistic

## Abstract

Seeking to improve personal armor equipment by providing mobility and resistance to penetration, this research aimed to explore the potential of sustainable materials in order to assess their ability in ballistic applications. Titica vine fibers (TVFs) extracted from aerial roots of *Heteropsis flexuosa* from the Amazon region were incorporated at 10, 20, 30, and 40 vol% into an epoxy matrix for applications in ballistic multilayered armor systems (MASs) and stand-alone tests for personal protection against high-velocity 7.62 mm ammunition. The back-face signature (BFS) depth measured for composites with 20 and 40 vol% TVFs used as an intermediate layer in MASs was 25.6 and 32.5 mm, respectively, and below the maximum limit of 44 mm set by the international standard. Fracture mechanisms found by scanning electron microscopy (SEM) attested the relevance of increasing the fiber content for applications in MASs. The results of stand-alone tests showed that the control (0 vol%) and samples with 20 vol% TVFs absorbed the highest impact energy (E_abs_) (212–176 J), and consequently displayed limit velocity (V_L_) values (213–194 m/s), when compared with 40 vol% fiber composites. However, the macroscopic evaluation found that, referring to the control samples, the plain epoxy shattered completely. In addition, for 10 and 20 vol% TVFs, the composites were fragmented or exhibited delamination fractures, which compromised their physical integrity. On the other hand, composites with 30 and 40 vol% TVFs, whose E_abs_ and V_L_ varied between 166–130 J and 189–167 m/s, respectively, showed the best physical stability. The SEM images indicated that for composites with 10 and 20 vol% TVFs, the fracture mode was predominantly brittle due to the greater participation of the epoxy resin and the discrete action of the fibers, while for composites with 30 and 40 vol% TVFs, there was activation of more complex mechanisms such as pullout, shearing, and fiber rupture. These results indicate that the TVF composite has great potential for use in bulletproof vests.

## 1. Introduction

Firearms are a persistent problem that has been escalating in large urban centers in emerging countries. In addition, armed conflicts still persist between nations and terrorist groups, creating a pressing need for the improvement of body armor systems to ensure the safety of combatants. In this regard, the search for new materials capable of absorbing high impact energies, as well as resisting penetration and promoting mobility, must be prioritized, taking into consideration the advancements in weapons and ammunition technology [1,2,3].

Multilayered armor systems (MASs) enable efficient personal protection against traumas caused by heavy ammunition. These systems consist of distinct layers of materials designed to withstand the impact of high-velocity projectiles (>800 m/s) [4,5,6]. A typical MAS features a ceramic front layer, which aims to fragment the projectile tip and absorb most of its energy [4,5,6,7,8,9]. The second layer is a lower-density material, often a polymer, that absorbs the residual energy of the ceramic fragments and projectile shrapnel resulting from the fragmented front layer [6,10]. Lastly, the third layer is commonly composed of a ductile metal [11] or, more recently, synthetic fabrics such as aramid [12,13].

The international standard NIJ 0101.04 [14], which assesses the ballistic resistance of personal body armor, provides two methods to determine the efficiency of a MAS. The first method involves measuring the trauma (indentation) caused in a clay witness backing after being shot with 7.62 mm × 51 mm NATO ammunition. The clay witness simulates the consistency of the human body and, according to the standard, the deformation caused by the projectile cannot exceed 44 mm, as anything beyond that could be considered lethal in real-life situations. The second method is based on a probabilistic approach that calculates the limit velocity (V_L_), i.e., the velocity above which the projectile can penetrate the armor and below which the projectile is stopped. The criterion used is the ballistic limit V50, which determines the velocity at which the probability of perforation would be 50% [3]. However, for high-velocity projectiles (>800 m/s), it is not feasible to calculate the V50 due to limitations in shooting at low speeds. As a result, it is not possible to guarantee non-perforation of the target, as the reduction in gunpowder reaches a limit where the projectile may not leave the firing device. In such cases, V_L_ is understood as the maximum velocity at which the target can absorb all the kinetic energy of the projectile, as described by Morye et al. [15].

Synthetic fabrics made of strong aramid fibers (e.g., Kevlar or Twaron), ultrahigh-molecular weight polyethylene (e.g., Dyneema), and polybenzoxazole (e.g., Zylon) have traditionally been used in MASs. However, in recent times, natural fiber-reinforced polymer composites (NFRPCs) have gained popularity as a cost-effective replacement for synthetic laminates in the second layer of a MAS [13,16,17,18,19,20,21]. Natural lignocellulosic fibers (NLFs), such as those derived from plant sources, offer several attractive properties, including low cost, light weight, minimal health risks during processing, good thermal and acoustic insulation characteristics, and reasonably good specific strength and modulus, as well as wide availability, among others [22,23,24]. As a result, NFRPCs have found numerous applications in areas such as civil construction panels [25], automotive parts [26], aeronautics [27], and, more recently, ballistic armor [28,29,30].

The fiber extracted from the *Heteropsis flexuosa*, a plant prevalent in the Amazon region, is also known as titica vine fiber (TVF). This NLF from the Araceae family [31], is rarely addressed in forestry studies [32]. In engineerable composites [33], TVF has shown results when incorporated into polymer matrices [33,34]. A recent study on TVFs revealed favorable properties for application in NFRPCs, describing them as comparable to many NLFs previously investigated in the literature for the same purpose, as compared in Table 1.

**Figure 1:**
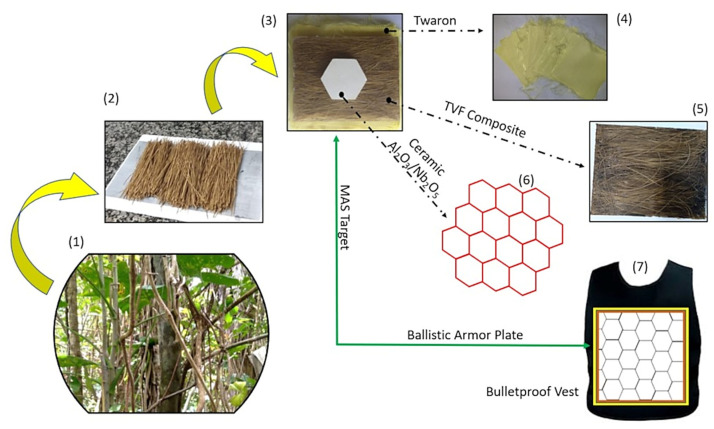
Components of the MAS used in the ballistic tests. (1) TVFs source plant; (2) TVFs; (3) MAS target; (4) Twaron fabric layers; (5) TVF/epoxy composite; (6) ceramic plates; and (7) bulletproof vest with the proposed armor plate.

According to Table 1, TVFs have one of the lowest densities among NLFs. In addition, the low microfibrillar angle and high crystallinity yield a comparable tensile strength for this fiber. These properties provided the motivation for a first-ever study on the use of TVFs in epoxy matrix composites both as the second layer of MASs and as stand-alone samples for the absorbed impact energy (E_abs_) and limit velocity (V_L_). The present work evaluated the ballistic performance of a MAS consisting of an Al_2_O_3_/Nb_2_O_5_ ceramic front layer followed by a composite plate, varying between 20 and 40 vol% TVFs, supported on a panel with 12 Twaron sheets that simulated a level IIIA ballistic vest (Figure 1). The depths of indentation were measured after the shots, which used 7.62 mm × 51 mm NATO ammunition. Stand-alone tests were also carried out on single composite plates (0 to 40 vol% of TVFs) to determine the E_abs_ after the impact and the V_L_ of the materials. The acquired data were statistically evaluated using the Weibull distribution, analysis of variance (ANOVA), and the Tukey test to identify behaviors and alterations in the failure mechanisms, as well as to investigate the level of reliability, significance, and equality of the results obtained.

## 2. Materials and Methods

### 2.1. Materials

TVFs were purchased at a local market in the city of Boa-Vista, in the state of Roraima, Brazil, and used as reinforcement for polymeric composites. Composite plates were made by means of compression molding using a commercial epoxy resin (E), diglycidyl ether of bisphenol A (DGEBA)-type, hardened with triethylenetetramine (TETA), in stoichiometric ratio phr 13, both supplied by Epoxy Fiber, Rio de Janeiro, Brazil. 

**Figure 2:**
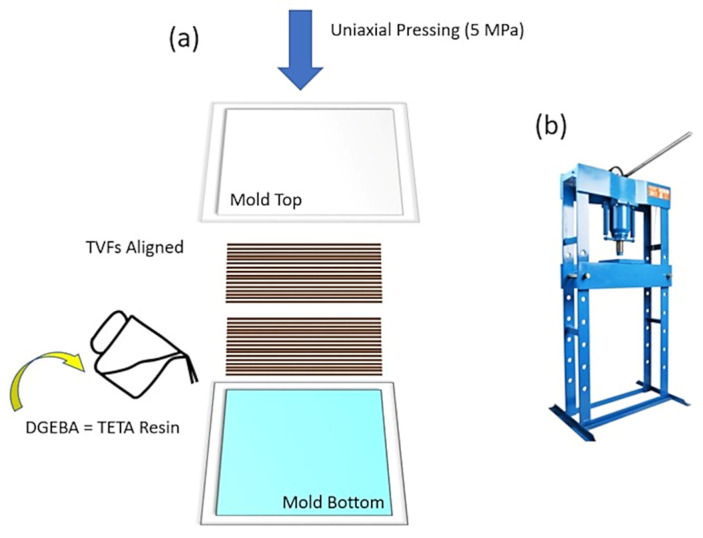
Manufacture of TVF/E boards. (**a**) Schematic of the compression molding process; (**b**) hydraulic press used for force application.

Before producing the composites, the fibers were cleaned in running water and dried in an oven at 60 °C for 24 h [33]. Then, composites with 0, 10, 20, 30, and 40 vol% TVFs, preferably aligned and incorporated into epoxy resin (TVF/E), were prepared using a hand lay-up process, in which a compression force of 5 MPa was applied in a Sky hydraulic press, São José do Rio Preto, Brazil, at room temperature (RT) for 24 h. The dimensions of the metallic mold used for processing were 150 × 120 × 11.9 mm. For the manufacture of composites, 0.50 g/cm^3^ was adopted as the average density for TVFs [33] and 1.11 g/cm^3^ for epoxy resin [45]. Figure 2 schematically presents the processing method used.

The production of the MAS targets involved the assembly of three layers: (i) a first layer of a 10 mm thick ceramic plate made of Al_2_O_3_ + Nb_2_O_5_; (ii) a second layer of a 10 mm thick TVF/E composite plate; and (iii) a third layer consisting of 12 sheets of Twaron. The Twaron fabric, which was supplied by the Teijin Aramid Company, Shanghai, China, was cut into rectangular sheets measuring 15 × 12 × 0.01 cm. The individual sheets were then bonded together using polyurethane glue. The Al_2_O_3_ + Nb_2_O_5_ ceramic plates were manufactured using a previously described procedure [46].

### 2.2. Methods

#### 2.2.1. Back-Face Signature (BFS) Tests of Multilayered Armor Systems (MAS)

**Figure 3:**
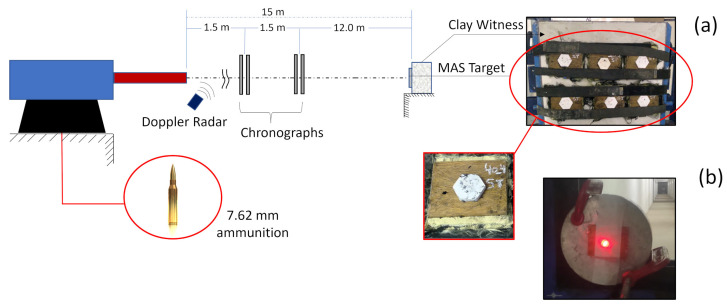
Schematic diagram of ballistic tests. (**a**) MAS target placement; (**b**) placement of the “stand-alone” sample.

The ballistic behavior of the composites was evaluated by means of two tests conducted at the Army Assessment Center (Caex) in Rio de Janeiro, Brazil. The first test involved placing a MAS target in front of a clay witness block supplied by Corfix, Porto Alegre, Brazil. After the shots were fired, a laser sensor (model Q4x Banner) was used to measure the back-face signature (BFS) depth of the resulting trauma. This test is commonly referred to as the BFS perforation and is described by the international standard NIJ 0101.04 [14]. The ballistic test was carried out as presented in Figure 3a.

Both BFS and stand-alone ballistic tests, the latter discussed below, used 7.62 mm caliber commercial ammunition, weighing 9.3 g, with metal coating. The velocity of the projectile before and after impact was measured using a radar model SL-520P Weibel Doppler, Alleroed, Denmark. For each proposed TVF volume content, five samples of MAS (20 and 40 vol% TVFs) were used.

#### 2.2.2. Stand-Alone Ballistic Tests

In the second test, as shown in Figure 3b, the individual performance of the TVF/E composite plates in terms of E_abs_ and V_L_ was assessed. This test is known as the stand-alone test. Seven independent composite plates (0, 10, 20, 30, and 40 vol% TVFs) were tested.

For stand-alone tests, the composite E_abs_ was estimated by the following equation:

(1) $$E_{abs}=\frac{M_{p}*\left(V_{i}^{2}-V_{r}^{2}\right)}{2}$$

where M_p_ is the mass of the projectile, V_i_ is the velocity of the projectile just before impact, and V_r_ is the residual velocity of the projectile after perforating the target.

The limit velocity (V_L_) is a dynamic parameter of great interest in materials for ballistic applications. Assuming that the target can fully stop the projectile, i.e., V_r_ is equal to zero, the limit velocity can be calculated with the equation:

(2) $$V_{L}=\frac{\sqrt{2E_{abs}}}{M}$$

where M is the mass of the projectile.

#### 2.2.3. Statistical Validation

The ballistic parameters, including BFS depth, E_abs_, and V_L_, were statistically evaluated and validated in terms of reliability and significance using the Weibull distribution and ANOVA, along with the Tukey test.

#### 2.2.4. Scanning Electron Microscopy (SEM)

Finally, the microscopic aspects of the ballistic impacted MASs and stand-alone TVF/E composite samples were analyzed using scanning electron microscopy (SEM), in a Quanta FEG 250 FEI microscope, Hillsboro, OR, USA, operating with secondary electrons and voltages up to 5 kV. All samples were sputter-coated with gold using the LEICA EM ACE600 equipment, Vienna, Áustria.

## 3. Results

### 3.1. Back-Face Signature (BFS) Tests of Multilayered Armor Systems (MAS)

**Figure 4:**
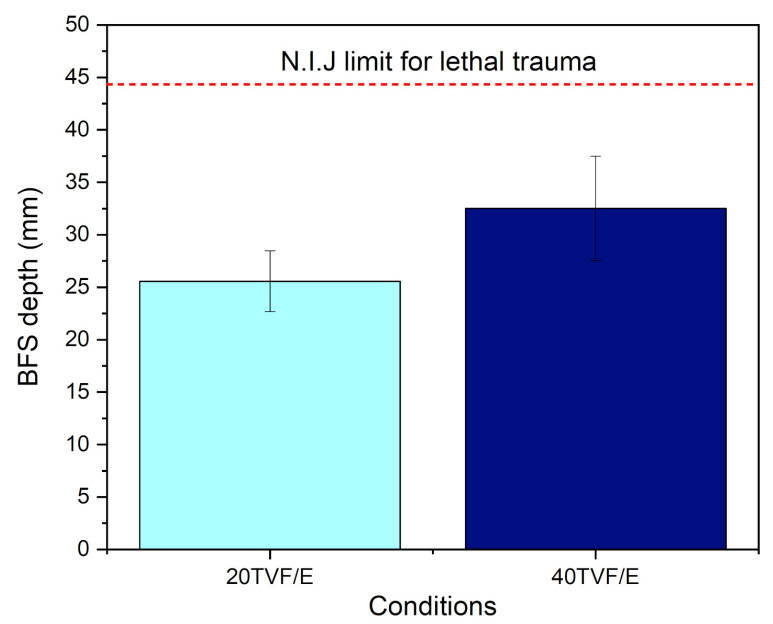
Back-face signature of the MAS using epoxy composites with 20 and 40 vol% TVFs incorporated as second layer.

Previous studies [47] confirm the efficiency of MASs consisting of three layers (ceramic + NLFs composite + aluminum alloy) that protect against level III ammunition and meet the BFS depth criteria established by the NIJ [14]. However, in the present work, armor plates were placed in front of target samples that simulated a level IIIA bulletproof vest to enhance their performance to level III protection against 7.62 mm ammunition. The results obtained for the MASs tested in this study are shown in Figure 4. The systems in which the intermediate composite plates have 20 and 40 vol% TVFs were denominated 20TVF/E and 40TVF/E, respectively. 

For all samples and conditions tested, there was no complete perforation of the target. In addition, for both groups, the BFS depth was less than 44 mm, the maximum limit allowed by the NIJ [14] to avoid lethal trauma. With the increase in the volume of fibers present in the second layer, the measured trauma increased from 25.57 to 32.51 mm. This same behavior was reported by other authors. Oliveira et al. [48] investigated the ballistic performance of both single composites of fique fabric and epoxy and their application as an intermediate layer in MASs. In their work, an increase in the BFS depth of 3.3 mm was verified when incorporating larger contents of fique fabric (15 to 50 vol%). Demosthenes et al. [49] verified that as the buriti fabric volume content was increased in epoxy matrix composites, there was a gradual increase in the BFS depths measured in the clay witness, with a jump from 18.9 mm for the 10 vol% samples to 25 mm for those with 30 vol%. This behavior is probably associated with transitions in the fracture mechanisms acting on the composites, which were evaluated by means of SEM and further discussed in a following separate section.

The Weibull distribution was used to quantify the statistical reliability of the measured BFS depth results. Table 2 presents the distribution parameters related to the evaluated property (BFS depth), where β is the Weibull modulus, θ the scale parameter, and R^2^ the coefficient of statistical precision.

Table 2 shows that the 40TVF/E composite had a lower β value, indicating a greater dispersion of values, as confirmed by the higher standard deviation of this group. The θ parameter followed the expected trend, correlating with the BFS depth measured in the test. Specifically, 62.3% of the tested samples in the 20TVF/E and 40TVF/E groups had a BFS depth of approximately 26.9 and 34.7 mm, respectively. The statistical precision coefficient R^2^ was highly representative and within an acceptable reliability range (>0.83). However, up to 17% of the samples in the 20TVF/E group and 4.5% in the 40TVF/E group could not be explained by the Weibull mathematical model.

**Figure 5:**
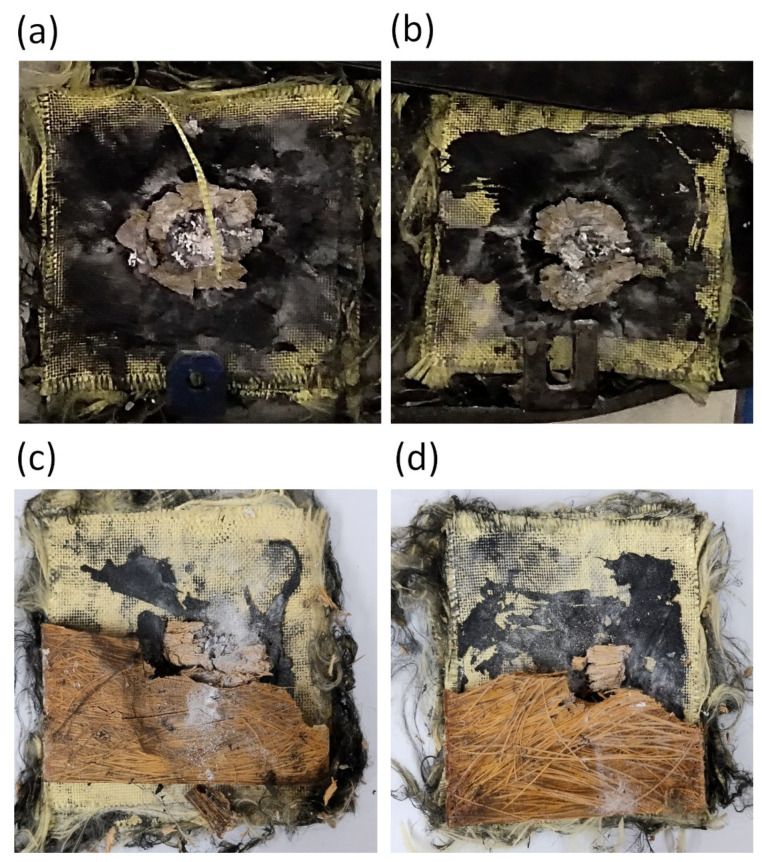
Frontal view of MAS target samples after ballistic testing: (**a**,**b**) 20TVF/E; (**c**,**d**) 40TVF/E.

Although the BFS depth increased with the volumetric fraction of TVFs, the standard deviations for the average measurements extracted from these groups may conceal the correlation owing to possible differences between the composites. To clarify this, ANOVA was performed on the results shown in Figure 4. The results indicate with 95% confidence that the values are different, as F_calc_ = 7.28 > F_critical_ = 5.31. Therefore, since there are only two sample conditions, it is possible to say that the volumetric fraction of fibers incorporated into the composites influenced the increase in the BFS depth exhibited by the MASs samples, with systems with 20TVF/E resisting the penetration of the projectiles better than those with 40TVF/E. Figure 5 depicts the appearance of the MAS target after being hit by a 7.62 mm projectile in ballistics tests using 20TVF/E and 40TVF/E as an intermediate layer.

In all samples the ceramic plate was completely destroyed (Figure 5). This occurs because this material is responsible for absorbing most of the impact energy [9]. Despite this, the composite layer plays a fundamental role in energy dissipation through the capture of ceramic fragments [19,50] that can be visualized by the presence of small white particles covering the fracture surface in the central area. This will be further explored by examining SEM images.

The ballistic tests showed that even though the MAS samples with an intermediate layer of 20TVF/E presented greater resistance to penetration due to their smaller BFS depth, the plates in this condition were almost completely fragmented, as seen in Figure 5a,b. In contrast, the targets with 40TVF/E showed traces of composite fragments stuck to the shield, indicating an improvement in the physical integrity of the material with an increase in fiber content, as shown in Figure 5c,d. This improvement in physical integrity is an important criterion for ballistic applications, as noted by Monteiro et al. [51]. 

**Figure 6:**
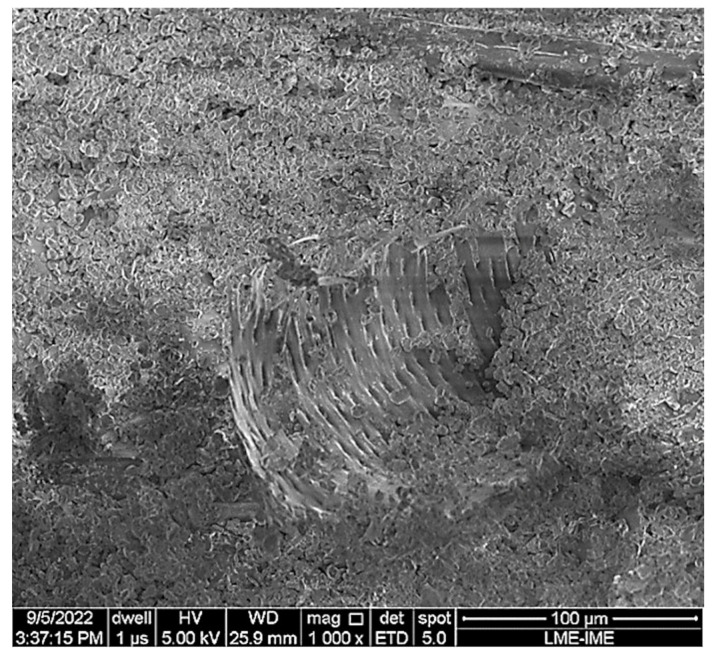
Fracture surface of the 20TVF/E composite illustrating ceramic fragments on the fiber.

The SEM image illustrated in Figure 6 shows the surface of a 20TVF/E composite covered by ceramic material. Note that the intermediate layer absorbed the kinetic energy of the fragments through mechanical encrustation. This is due to electrostatic charges and van der Walls forces acting on the surface of the composite [11].

**Figure 7:**
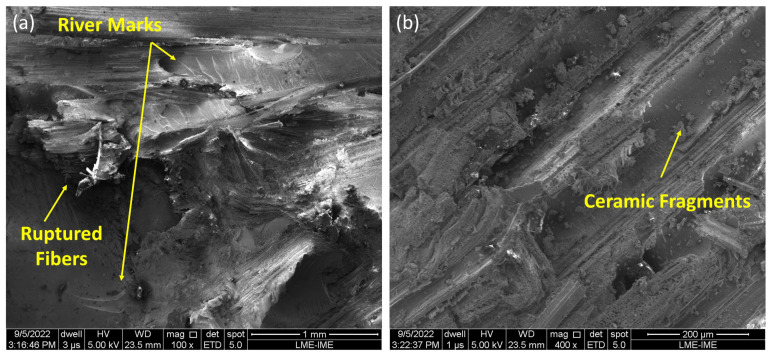

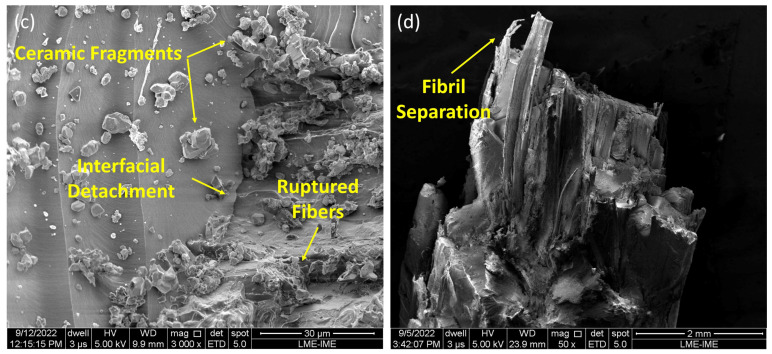
Fracture surface aspect of the different composite formulations: (**a**,**b**) 20TVF/E; (**c**,**d**) 40TVF/E.

To better understand the fracture and energy absorption mechanisms at work, SEM images were taken on the surface of the target samples after the 7.62 mm shootings, as shown in Figure 7.

The performance of the target MASs can be explained through an evaluation of the fracture surface of these samples. Figure 7a,b confirms the brittle fracture tendency of the 20TVF/E composites. Although mechanisms of fracture and energy dissipation are observed, such as TVFs rupture and the capture of ceramic fragments, the micrographs point to the strong influence of the epoxy resin, which is predominant throughout the composite. In addition, there is a significant presence of river marks, associated with crack propagation.

When checking the images in Figure 7c,d, mechanisms similar to those evidenced in the 20TVF/E samples are also present. However, others can still be identified, such as interfacial detachment and fibril separation. These same mechanisms of energy dissipation were reported by Costa et al. [12] when evaluating the ballistic performance of epoxy composites reinforced with curaua fibers.

The 40TVF/E samples exhibited more complex mechanisms due to the action of TVFs, as evidenced by a rougher fracture surface and better physical integrity. However, the greater BFS depth after impact on the MASs samples with 40TVF/E larger fiber volumes suggests that these mechanisms were not sufficient to improve the ballistic behavior. This could be attributed to weak compatibilization at the TVF/E interface, which is often associated with the hydrophobic nature of the matrix and the hydrophilic nature of NLFs. Additionally, the presence of impurities on the surface of the TVFs such as waxes and oils can make it difficult to anchor with the matrix. Furthermore, TVFs have lower mechanical properties compared to the epoxy resin, which has a tensile strength of 38 MPa and an elastic modulus of 1.38 GPa [33], whereas TVFs have 26 MPa and 1.02 GPa, respectively, as described elsewhere [52].

Table 3 compares the materials used as an intermediate layer in MAS available in the literature and those investigated in the current work.

According to the data presented in Table 3, it is observed that the results of the composites with 20 vol% of TVFs are similar to those obtained using traditionally known NLFs, such as buriti and coir, reinforcing the intermediate layer of MASs. Increasing the TVF fraction to 40 vol% leads to BFS depth measurements closer to those obtained with 30 vol% of coir and guaruman fibers, indicating that higher TVF fractions are more effective in enhancing ballistic performance. Interestingly, the MASs with 40TVF/E exhibit superior ballistic performance compared to those with bagasse/E (at a lower fraction) and a Dyneema plate. It should be noted that the systems with bagasse and coconut fibers used an aluminum plate (ρ_al_ = 2.66 g/cm^3^) [53] as the third layer, while in this study, 12 sheets of Twaron fabric (ρ_Twaron_ = 1.44 g/cm^3^) [54] were used, resulting in a lighter and more mobile individual.

### 3.2. Stand-Alone Ballistic Tests

Table 4 presents the results of V_i_ and V_r_ that enabled the calculations of the E_abs_ and V_L_, according to Equations (1) and (2), for the stand-alone tests with 7.62 mm ammunition.

From the values calculated for E_abs_ and V_L_, the Weibull distribution was performed to determine the degree of dispersion and the level of precision associated with the ballistic results obtained, as shown in Table 5.

Based on the results shown in Table 4 and Table 5, higher values of E_abs_ and V_L_ can be noted for the 0TVF/E (plain epoxy) condition. In general, the data show a downward trend as the content of TVFs increases in the composite. This behavior can be explained by the brittle characteristic of the epoxy matrix, which tends to dissipate more energy by generating fracture surfaces [55,56]. Garcia Filho et al. [56] noticed a decrease of 80 J in the energy absorbed by the 40 vol% compared to the 10 vol% fiber-reinforced composites when investigating the ballistic potential of epoxy composites with piassava fibers. Similarly, Pereira et al. [57] reported in their research the same decreasing trend with the increase in the volumetric fraction of fique fibers and fabric inserted in a polyester matrix.

According to the Weibull distribution, it is possible to infer that out of the seven samples tested for each condition, four will present E_abs_ and V_L_ with values close to the θ calculated for their respective group. Additionally, the 20TVF/E group stands out as the least homogeneous of all the conditions evaluated. This can be better explained by the low β obtained, which characterizes a less narrow distribution, i.e., with little repeatability of the results. It is suggested that this fact is related to processing defects, such as the presence of voids and bubbles acting as stress concentrators and faults. In addition, the R^2^ correlation coefficient showed good adjustment (>0.85), therefore demonstrating high data accuracy.

According to Table 6, in view of the results of the ANOVA of the E_abs_ and V_L_, it can be stated with a confidence level of 95% that the results are statistically different, since F_cal_ > F_critical_.

To determine the significance of the differences in mean values between the treatments, a Tukey test was performed, and the results are presented in Table 7. With five treatments and 30 degrees of freedom, the studentized amplitude (q) was calculated to be 4.1. From this, an honestly significant difference (HSD) of 34.62 J for E_abs_ and 19.71 m/s for V_L_ was determined. The mean values that are significantly different from the others are highlighted in Table 7.

The comparison between the averages presented in Table 7 indicates that plain epoxy demonstrated the best performance in terms of energy absorption compared to the other conditions. Although the group with 20 vol% showed slightly better performance among those with some percentage of incorporated fiber, these were statistically equal to those with 10 and 30 vol% since the calculated difference between the means was smaller than the HSD. Formulations with 0, 10, 20, and 30 vol% were found to be statistically different from the 40 vol% condition. It can be inferred that this is related to the relevant performance of the epoxy resin, which is present in greater quantity in these samples. Moreover, the fact that it has higher mechanical properties than TVFs explains the higher mean values calculated for the 0 and 40 vol% (Table 7) and the sharp decrease in absorbed energy observed in Table 4. In terms of V_L_, the comparison between the averages shows a similar trend.

**Figure 8:**
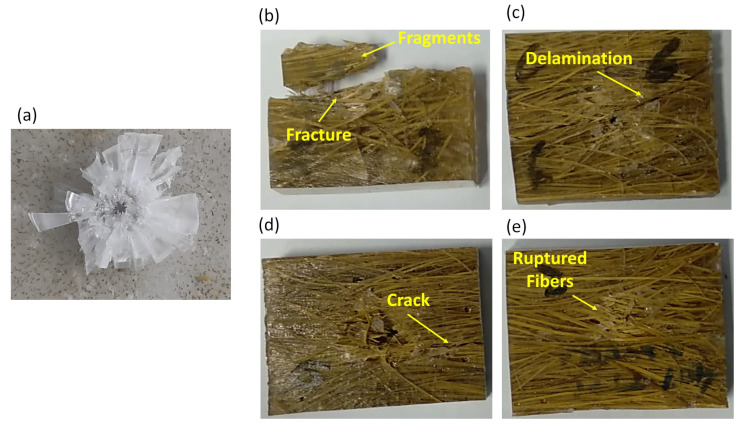
Macroscopic analysis of the samples after the stand-alone ballistic test using ammunition 7.62 mm: (**a**) pure epoxy; (**b**) 10TVF/E; (**c**) 20TVF/E; (**d**) 30TVF/E; (**e**) 40TVF/E.

Once again, the physical integrity of the materials must be considered. On the one hand, the plain epoxy and samples with higher epoxy matrix percentages showed higher E_abs_ and V_L_ values, but on the other hand, they either shattered completely or had long cracks propagating throughout, compromising their physical integrity. In contrast, increasing the content of TVFs attenuated such problems. In fact, materials for ballistic shielding application should not be destroyed after the first shooting and must be able to support other impacts and continue to dissipate energy after several shootings. Used as an intermediate layer in a MAS, a plain epoxy plate would not reach this requirement. Figure 8 presents composite plates with 0, 10, 20, 30, and 40 vol% of TVFs after the ballistic impact of a 7.62 mm projectile. 

Analysis of the post-shooting impact aspects shows the complete fragmentation of the plain epoxy plate (Figure 8a). After incorporating a small amount of TVFs (10 vol%) this effect is attenuated, but the partial fragmentation of materials is still observed (Figure 8b). With the insertion of 20 vol% of TVFs, the composite has greater dimensional stability compared to the two previous conditions. However, it is possible to notice the presence of cracks that have propagated from end to end in the material (Figure 8c). For condition 30TVF/E, the samples display smaller cracks, and their propagation is interrupted due to the greater performance of the TVFs (Figure 8d). The sample that stood out in terms of physical integrity was the 40TVF/E (Figure 8e). In the tests of this condition, only the presence of broken fibers in the region where the shooting occurred can be highlighted, with the absence of cracks and deformations on the surface of the material that could be seen macroscopically. Therefore, this condition showed the best potential for ballistic applications as an intermediate layer. Although it did not have the best results for E_abs_ and V_L_, its integrity was preserved. In addition, when employed in conjunction with other materials that constitute an MAS, it proved its efficiency by presenting a BFS depth below the limit established by the NIJ standard of 44 mm [14]. 

**Figure 9:**
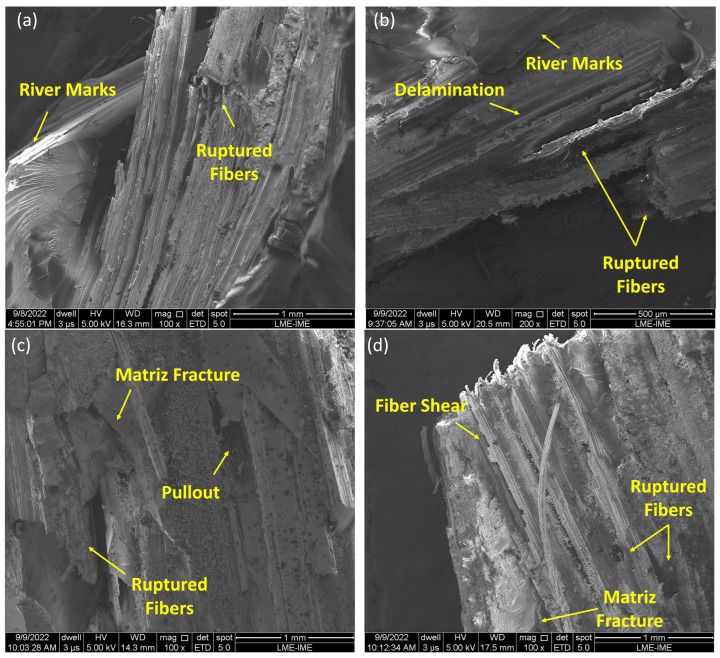
Microscopic analysis of the fracture surface of composites after stand-alone tests: (**a**) 10TVF/E; (**b**) 20TVF/E; (**c**) 30TVF/E; (**d**) 40TVF/E.

Composite samples of 10, 20, 30, and 40 vol% of TVFs exhibited failure mechanisms that were specifically verified after fractographic analysis by SEM. The images obtained are illustrated in Figure 9.

When evaluating the behavior presented by the 10TVF/E and 20TVF/E composites (Figure 9a,b), several common failure mechanisms are identified. These samples obtained the highest E_abs_ values after the impact of the projectile. This characteristic was associated with a greater performance of the brittle fracture mechanisms of the epoxy resin. It was evidenced by the strong presence of river marks. Despite this, the discreet action of the fibers can be visualized by the appearance of broken fibers and delamination on the surface of the material. Thus, when considering the post-fracture aspects presented by these two groups, it is possible to perceive that the corresponding TVF contents were not efficient in these fractions, as they did not induce a change in behavior, a predominantly brittle fracture, or the triggering of more complex fracture mechanisms. 

When observing Figure 9c,d, it is noted that the areas close to the occurrence of the shooting became more rugged, and analysis of the mechanisms present was difficult. However, the absence of both river marks and the propagation of long cracks on the surface of the material is noticeable. In addition, mechanisms such as pullout, shearing, and fiber rupture are more frequent in the micrographs, giving evidence that more complex fracture mechanisms were activated and those that already existing were intensified, despite the fracture mode being persistently brittle.

Even with the inferior ballistic performance of the 30TVF/E and 40TVF/E samples, the action of the TVFs is quite evident in the images, being efficient in the sense of promoting barriers against the propagation of cracks and fissures and, consequently, retaining the integrity of the material. On the other hand, it is inefficient in terms of raising the E_abs_ by the plate.

Table 8 shows a comparison of E_abs_ and V_L_ for the TVFs and epoxy composites tested as a stand-alone and previous results available in the literature, in which several NLFs were used, including some that were incorporated into polyester resin. 

When evaluating the ballistic performance of the TVFs and epoxy composites, it is noticeable that these presented E_abs_ and V_L_ values higher than any other composites of polyester matrix. These include the plates with 30 vol% curaua and epoxy as well as the aramid fabric, all with the same thickness of 10 mm, as shown in Table 8. Furthermore, the composites with 20 and/or 30 vol% TVFs are comparable to buriti fabric [49] and *C. malaccensis* fibers [18], both incorporated in an epoxy matrix. It is also worth noting that the composites with buriti fabric and *C. malaccensis* fibers showed higher standard deviations compared to those in the present work. This indicates a greater homogeneity of samples with TVFs. 

The greatest decay of the parameters listed in Table 8 occurs in samples of 30 vol%. The condition with 40 vol% TVFs exhibited 62 J less compared to piassava in the same fraction of fiber and resin. A portion of this result can be explained by poor fiber/matrix adhesion and pressing with excessive load during sample processing, which may have enabled the generation of microcracks in the material, but, as previously mentioned, the main factor is that the mechanical properties of TVFs are lower than those of the epoxy matrix. Despite this, when applied to MASs, none of the samples underwent total perforation, and all showed a BFS depth smaller than 44 mm, with the 40TVF/E condition that maintained the physical integrity of the composite. Therefore, together with the 30TVF/E, it is suggested that 40TVF/E is the most efficient for applications in ballistic protection.

## 4. Summary and Conclusions

For the first time, composites with titica vine fibers (TVFs) from the Amazon incorporated into a polymeric epoxy matrix were ballistically tested. In both ballistic tests, i.e., back-face signature (BFS) and stand-alone, these materials revealed promising results for application in personal protection vests.

The multilayered armor system (MAS) targets, with an intermediate layer composed of composites containing 20 and 40 vol% TVFs, presented a BFS depth in the clay witness of 25.6 mm and 32.5 mm, respectively. Both values were well below the maximum limit of 44mm established by the NIJ standard. Unlike the 20 vol% condition, where the composite almost completely fragmented, the samples with 40 vol% of TVFs exhibited better integrity after impact, as recommended by the standard.

The investigation of micrographs obtained by performing scanning electron microscopy (SEM) on MAS targets with a 20 vol% TVF composite after fracture showed the predominance of brittle epoxy fractures and the presence of fracture mechanisms and energy dissipation such as fiber rupture and the capture of ceramic fragments. Upon increasing the percentage to 40 vol%, additional features such as interfacial detachment and fibril separation were observed using SEM.

Composite samples with fiber volume fractions ranging from 0, 10, 20, 30, and 40 vol% were tested through stand-alone ballistics tests. The results showed a decreasing trend in the absorbed impact energy (E_abs_) and limit velocity (V_L_) parameters with increasing fiber volume fraction in the matrix. The values of E_abs_ ranged from 212 to 131 J, and the V_L_ values ranged from 213 to 167 m/s. Despite this decline, the plates with 30 vol% of TVFs showed superior performance when compared to polyester composites reinforced with fique and sisal fibers. Furthermore, the epoxy composites with 40 vol% of TVFs outperformed the aramid fabric ply and curaua fibers (30 vol%) in an epoxy matrix.

The plates with 30 and 40 vol% of TVFs showed better dimensional stability after the shootings and stood out in comparison to other materials available in the literature. Even though the matrix exhibited brittle fractures, failure mechanisms such as pullout, shear, and fiber ruptures were observed in the fibers, and these mechanisms were efficient in preventing the propagation of cracks and fissures on the material surface and maintaining its physical integrity. However, they were not as efficient in terms of E_abs_ and V_L_ as seen previously, but their measurements were comparable and even superior to those of other materials reported in the literature.

## Figures and Tables

**Table 1:** Reported properties for TVFs when compared to other NLFs.

Fiber	Apparent Density (g/cm^3^)	Microfibrillar Angle (Degree)	Crystallinity Index (%)	Tensile Strength (MPa)	Young’s Modulus (GPa)	Reference
Titica vine	0.50 ± 0.07	7.95	78	25.92 ± 6.69	1.02 ± 0.22	[33]
*Catharanthus roseus*	1.34 ± 0.57	-	25.09	27.02 ± 1.1	1.23 ± 0.04	[35]
Coir	1.20 ± 0.24	30–49	43.50	44.0 ± 8	2.0 ± 0.3	[35,36,37]
*Tridax procumbens*	1.16 ± 0.12	13.4	34.46	25.75 ± 2.45	0.94 ± 0.09	[38]
*Perotis indica*	0.79	8.45–15.87	48.3	32.3	69.61	[39,40]
Sugar cane	1.28	-	35	169.51 ± 18.65	5.18 ± 0.63	[41,42]
*Parthenium hysterophorus*	1.25	-	40.68	24 ± 2	-	[43]
Aerial roots of banyan tree	1.23	10.88	72.47	19.37 ± 7.72	1.8 ± 0.40	[44]

**Table 2:** Weibull parameters for the BFS depth of the samples.

Sample	BFS (mm)	β	ϴ	R^2^
20TVF/E	25.57 ± 2.89	8.725	26.92	0.8347
40TVF/E	32.51 ± 4.98	6.509	34.73	0.9559

**Table 3:** Comparison between the BFS depths exhibited by natural fiber-reinforced epoxy composites (10 mm thick) and a Dyneema plate (25 mm thick) for application as second layer in MASs.

Material in Ballistic Armor for Protection against Level III Ammunition	BFS (mm)	Last Layer	Reference
20TVF/E	25.57 ± 2.89	Twaron	PW
40TVF/E	32.51 ± 4.98	Twaron	PW
20Buriti/E	21 ± 1.9	Al Plate	[49]
20Coir/E	22 ± 2	Al Plate	[10]
30Coir/E	31.6 ± 2.7	Al Plate	[10]
30Guaruman/E	32.9 ± 1.6	Kevlar	[13]
30Sugarcane bagasse/E	39 ± 8	Al Plate	[53]
Dyneema plate	41.5 ± 1.8	Kevlar	[16]

**Table 4:** Parameters and results from the stand-alone ballistic tests with 7.62 mm ammunition for the epoxy composites reinforced with TVFs.

Sample	V_i_ (m/s)	V_r_ (m/s)	E_abs_ (J)	V_L_ (m/s)
0TVF/E	814.40 ± 8.54	785.96 ± 7.01	211.69 ± 21.56	213.27 ± 6.94
10TVF/E	805.55 ± 8.45	782.28 ± 7.89	171.82 ± 15.88	192.06 ± 8.80
20TVF/E	813.91 ± 4.69	790.28 ± 6.95	176.19 ± 31.41	193.88 ± 17.75
30TVF/E	820.17 ± 4.16	797.74 ± 4.94	166.51 ± 23.36	188.84 ± 13.08
40TVF/E	815.68 ± 11.14	798.26 ± 12.69	130.53 ± 19.50	167.14 ± 12.60

**Table 5:** Weibull parameters for absorbed energy and limit velocity of the samples.

	Sample	β	ϴ	R^2^		Sample	β	ϴ	R^2^
E_abs_	0TVF/E	15.98	218.1	0.9354	V_L_	0TVF/E	32.02	216.6	0.9373
	10TVF/E	11.28	179.1	0.9040		10TVF/E	22.55	196.3	0.9035
	20TVF/E	5.268	190.9	0.9215		20TVF/E	10.53	202.6	0.9212
	30TVF/E	7.292	177.1	0.8538		30TVF/E	14.58	195.1	0.8533
	40TVF/E	6.599	139.6	0.8619		40TVF/E	13.21	173.3	0.8624

**Table 6:** ANOVA of the absorbed energy and limit velocity of composites from 0–40 vol% TVFs.

Variation Causes	E_abs_					V_L_				
	DF	SS	MS	F_calc_	F_critical_	DF	SS	MS	F_calc_	F_critical_
Treatment	4	23,385.50	5846.38	11.71	2.69	4	7555.31	1888.83	11.68	2.69
Residue	30	14,977.49	499.25			30	4851.86	161.73		
Total	34	38,362.99				34				

**Table 7:** Results obtained for the differences (HSD) between the average values of the impact E_abs_ and the V_L_, in the volumetric fractions of 0, 10, 20, 30, and 40 vol% TVFs, after the Tukey test.

Sample	E_abs_					V_L_				
	0TVF/E	10TVF/E	20TVF/E	30TVF/E	40TVF/E	0TVF/E	10TVF/E	20TVF/E	30TVF/E	40TVF/E
0TVF/E	0	39.87	35.50	45.19	81.16	0	21.22	19.40	24.43	46.13
10TVF/E	39.87	0	4.37	5.32	41.29	21.22	0	1.82	3.21	24.92
20TVF/E	35.50	4.37	0	9.68	45.66	19.40	1.82	0	5.03	26.74
30TVF/E	45.19	5.32	9.68	0	35.98	24.43	3.21	5.03	0	21.70
40TVF/E	81.16	41.29	45.66	35.98	0	46.13	24.92	26.74	21.70	0

**Table 8:** Comparison of E_abs_ and V_L_ for the composite plates reinforced with titica vine fibers and other composites with incorporated natural fibers, as well as the aramid fabric plate.

Conditions	Matrix	E_abs_ (J)	V_L_ (m/s)	Reference
TVF—0%	Epoxy	211.69 ± 21.56	213.27 ± 6.94	PW
TVF—10%	Epoxy	171.82 ± 15.88	192.06 ± 8.80	PW
TVF—20%	Epoxy	176.19 ± 31.41	193.88 ± 17.75	PW
TVF—30%	Epoxy	166.51 ± 23.36	188.84 ± 13.08	PW
TVF—40%	Epoxy	130.53 ± 19.50	167.14 ± 12.60	PW
Fique fiber—20%	Polyester	121 ± 11	−	[57]
Fique fiber—30%	Polyester	113 ± 4	−	[57]
Fique fabric—20%	Polyester	156 ± 12	−	[57]
Fique fabric—30%	Polyester	97 ± 7	−	[57]
Buriti fabric—20%	Epoxy	178 ± 54	190 ± 30	[49]
Buriti fabric—30%	Epoxy	189 ± 50	194 ± 97	[49]
*C. malaccensis*—20%	Epoxy	222.11 ± 22.38	213.74 ± 10.56	[18]
*C. malaccensis*—30%	Epoxy	167.18 ± 39.05	184.40 ± 21.58	[18]
Piassava fiber—40%	Epoxy	192 ± 13	198 ± 6	[56]
Sisal fiber—20%	Polyester	116	−	[58]
Sisal fiber—30%	Polyester	139	−	[58]
Curaua—30%	Epoxy	106 ± 11	−	[11]
Aramid fabric	−	58 ± 29	109 ± 7	[11]

## Data Availability

Not applicable.
